# Institutionalizing Open Science in Africa: Limitations and Prospects

**DOI:** 10.3389/frma.2022.855198

**Published:** 2022-04-15

**Authors:** Izuchukwu Azuka Okafor, Smart Ikechukwu Mbagwu, Terkuma Chia, Zuwati Hasim, Echezona Ejike Udokanma, Karthik Chandran

**Affiliations:** ^1^Department of Anatomy, Faculty of Basic Medical Sciences, College of Health Sciences, Nnamdi Azikiwe University, Nnewi, Nigeria; ^2^Department of Obstetrics and Gynaecology, College of Medicine, University of Ibadan, Ibadan, Nigeria; ^3^Pan African University of Life and Earth Science Institute (Including Health and Agriculture), University of Ibadan, Ibadan, Nigeria; ^4^Beth Israel Deaconess Medical Centre, Harvard Medical School, Boston, MA, United States; ^5^Department of Anatomy, Faculty of Basic Medical Science, College of Health Sciences, Nile University, Abuja, Nigeria; ^6^Department of Language and Literacy Education, Faculty of Education, University of Malaya, Kuala Lumpur, Malaysia; ^7^Birmingham Business School, University of Birmingham, Birmingham, United Kingdom; ^8^Department of Automation, Shanghai Jiao Tong University, Shanghai, China

**Keywords:** open science, Africa, advocacy, institutions, engagement

## Abstract

The advancement of scientific research and raising the next-generation scientists in Africa depend largely on science access. The COVID-19 pandemic has caused discussions around open science (OS) to reemerge globally, especially in resource-poor settings like Africa, where the practice of OS is low. The authors highlighted the elements, benefits, and existing initiatives of OS in Africa. More importantly, the article critically appraised the challenges, opportunities, and future considerations of OS in Africa. Addressing challenges of funding and leadership at different levels of educational, research, and government parastatals may be pivotal in charting a new course for OS in Africa. This review serves as an advocacy strategy and an informative guide to policymaking and institutionalization of OS in Africa.

## Introduction

**Figure 1:**
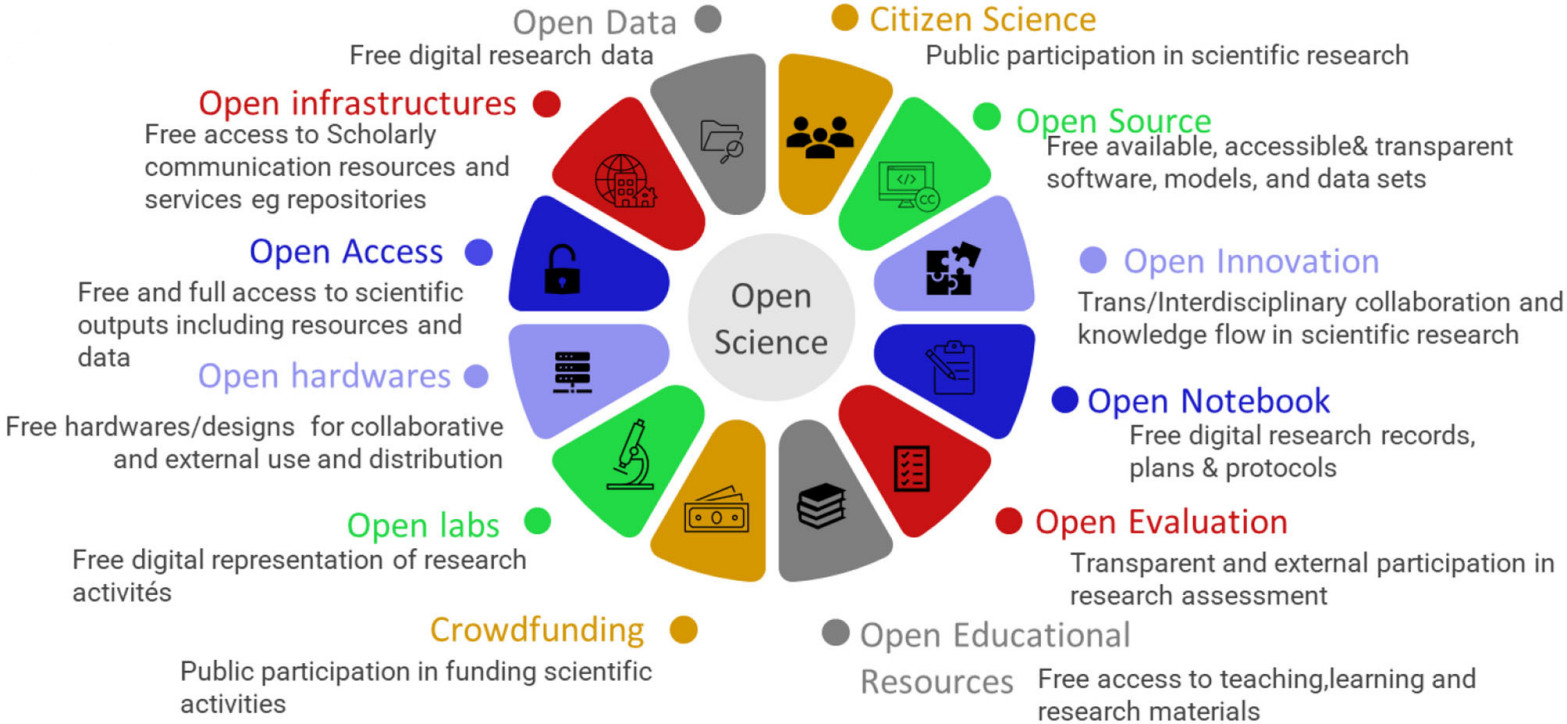
The elements of open science as adapted from the UNESCO recommendation 2021.

Open science (OS) is a movement focusing on making science more open, accessible, effective, democratic, and transparent to society, notwithstanding the level of education (1). Suffice to say that OS, as an inclusive science, potentially closes the science, technological and innovation divide between and within nations. According to the final report of the United Nations Educational, Scientific and Cultural Organization (UNESCO) on OS, twelve elements of OS exist, including open data, open infrastructure, open access (OA), open hardware, open laboratories, open-source, open innovation, open notebook, open evaluation, open educational resources (OERs), crowd funding, and citizen science (UNESCO, 2021). UNESCO has described these elements of OS in their recent recommendations (UNESCO, 2021). The recommendations has posited that none of the elements of OS should be neglected in implementing OS strategies and all the components should work in synergy to produce a more effective and scalable OS system. These elements have been summarized in Figure 1.

Access to science holds the key to strengthening health systems, advancing scientific research, and efficiently training Africa's next-generation scientists. However, this access is limited mainly due to inadequate funding of science in Africa, with poor funding of 0.1–0.5% gross domestic product (GDP) for science and technology in many African countries as against UNESCO's recommendation of at least 1% GDP (Christie, 2019; Krishna, 2020). Since the COVID-19 pandemic, the need for OS has re-emerged in Africa and other resource-poor settings because of its critical role in pandemic preparedness and response. The inequality in OS practices and the associated consequences in Africa compared to the developed countries became more evident with the planning and implementation of COVID-19 pandemic responses (Havemann et al., 2020).

OS as a phenomenon is either misconstrued, neglected, or not yet institutionalized (Krishna, 2020). The late sixteenth and early seventeenth century saw the emergence of OS (David, 2004). Modern OS was started by the global adoption of the institutional scientific journal. Consequently, England established the Royal Society in 1660 while France established the French Academy of Sciences in 1666 (David, 2004). The 1990s OS movement began in the United States of America as a springboard to its global spread (Venith, 2015). Following the 2015 competitiveness council, the European Research Ministers developed European OS Agenda (Heise and Pearce, 2020). This has seen the commencement of diverse OS and OA projects and initiatives like OpenAIRE, RECODE, and OpenScienceLink (Heise and Pearce, 2020). Africa has witnessed some OS projects, including Africa Open Science Platform (AOSP), DataFirst, and OA for Africa. Further, Library Support for Embedded NREN Services and E-infrastructure (LIBSENSE), which supports OS and research in Africa, was launched in 2017 with diverse regional workshops conducted (Table 1) (Kuchma, 2022). Despite these efforts, only three African nations (Gabon, Mauritius, and Namibia) gave written feedback to the first draft of the Recommendation on OS out of the 47 UNESCO Africa member states (UNESCO, 2021). These questions the political will of the African member states to fully institutionalize OS in their countries. Kenya, Ghana, and Morocco first embraced the OS movement. While French-speaking Sub-Saharan African nations have shown hesitation in adopting this movement, Burkina Faso and Sierra Leone led OS adoption in this region. This movement notwithstanding, it took the current COVID-19 to strongly re-establish the need for OS (UNESCO, 2021). Following the globally adopted public health measures in containing COVID-19, there is a need for real-time data on the rate of infection, mortality, and emerging variants in nations. This is pertinent in constantly evaluating how nations are faring in disease containment, treatment, and lessons drawn for worse hit nations. This strategy re-instated the need for OS globally, especially in Africa where it has been underutilized. In this critical time, lessons may be drawn from most African nations with minimal infection and mortality rates; however, the dearth of OS may have been a limitation.

**Table 1:** Some open science initiatives/platforms in Africa.

**Projects/initiatives**	**Hosts/country**	**Type**	**Focus**	**Period/year of establishment**	**Remarks**
Africanfossils.org (https://africanfossils.org/)	Partnership by Autodesk, Turkana Basin Institute, and the National Museums of Kenya, Stony Brook University and the National Geographic Society.	Virtual lab for fossil collections	• To host a collection of 3D models of significant fossils and artifacts for researchers students and interested audience	2014	Promotes the increase in knowledge to the public on prehistoric times
African Virtual University Project (AVU) (https://avu.org/avuweb/)	Pan African Intergovernmental Organization with headquarters in Kenya	eLearning Network	• To provide education in the area of Science, Renewable Energy, Food Security and ICT, etc.	1997	Provides educational training to 18 participating African countries
African Journals Online (https://www.ajol.info/index.php/ajol)	South Africa	Digital Repository For African research	• To increase global and continental online access, awareness, quality and use of African-published, peer-reviewed research.	1998	Currently hosts 535 Journals with 274 Open Access Journals
The Scholarly Communication in Africa programme (SCAP) (http://www.cilt.uct.ac.za/cilt/scap)	Centre for Educational Technology and the Research Office at the University of Cape Town. In close collaboration with the Southern African Regional Universities' Association (SARUA),	Training funded by Canadian International Development Research Centre (IDRC)	• To increase African universities' contribution to regional and global knowledge production.	2010–2014	Promoted the visibility of African researchers, creation of repositories and exploration of affordable business models for the open online publication of scholarly materials
Open Access for Africa (https://umb.libguides.com/OAA)	UNESCO and the Network of African Science Academies (NASAC), Royal Netherlands Academy of Arts and Sciences, Kenya National Academy of Sciences, African Academy of Sciences, and Kenyan Ministry of Education, Sciences and Technology.	Advocacy	• Provision of expert intervention for research and development in Africa.	2015 (29–30 January)	UNESCO encouraged the establishment of training centers for capacity building in the area of open Access philosophies and systems.
African Digital Research Repositories (https://www.internationalafricaninstitute.org/repositories)	International African Institute (IAI), London and AfricArXiv	Digital repository	• Improve the discoverability of African research and publications • Enhance the interoperability of existing and emerging African repositories • Identify ways through which digital scholarly search engines can enhance the discoverability of African research	2016	Promotes research-based knowledge from African repositories
Electronic Publishing (https://codesria.org/spip.php?rubrique257andlang=en)	Council for the Development of Social Science Research in Africa (CODESRIA), Dakar, Senegal.	Advocacy	• To discuss opportunities and challenges to the Open Science movement in the region.	2016 (March 30–April 1)	Dakar Declaration on Open Science in Africa to promote and support Open Science across Afsrica.
The African Open Science Platform (AOSP) (https://council.science/current/news/the-national-research-foundation-of-south-africa-to-host-the-african-open-science-platform/)	National Research Council of South Africa supported by South Africa's Department of Science and Innovation (DSI), key institutions in Africa, and the International Science Council (ISC).	Advocacy	• To provide current landscape of data/science initiatives in Africa • To create a Pan-African open science community. • To promote the formation of a national open science fora.	2016 (Operational kick off in 2020)	Encourages increased commitment to Open Science
LIBSENSE (Library Support for Embedded NREN Services and e-infrastructure) (https://spaces.wacren.net/display/LIBSENSE/Home)	WACREN—West and Central African Research and Education Network in partnership with different organizations.	Repositories	• Advancing open Science in Africa through strengthening and expanding services at the institutional, national and regional level.	2017	Promotes the availability and adoption of indigenous open science services and infrastructures in Africa
AfricArXiv (https://info.africarxiv.org/)		Digital archive for African research,	• Provide open access to research information • Highlight, display and promote African journals and African research output and expertise • Provide collaboration among African scientists locally and globally. • Fill the gaps where institutional repository systems are missing	2018	Provides platform for preprints, accepted manuscripts (post-prints), and published articles of African scientists. Provide collaboration among African scientists.
The H3ABionet project (https://www.h3abionet.org/)	South Africa	Bioinformatics Network	• Education and training • Development of Bioinformatics tools and services • Scientific engagement and communications	2019	Provides Support for research in genomic sciences
African Academy of Sciences (AAS) Open Research (https://aasopenresearch.org/)	Headquarters is located in Kenya	Repository	• For publication and peer review of research articles majorly supported by AAS and The Alliance for Accelerating Excellence in Science in Africa (AESA)	2019	Provides scholarly impact while promoting reproducibility and transparency
DataFirst (https://www.datafirst.uct.ac.za/)	South Africa	Data Repository	• Provides a repository of data for South Africa • Provides training and research on data quality and usage	2020	Promotes access to open research data infrastructure especially in South Africa

OS is undoubtedly beneficial but embodies diverse challenges in Africa, may involve some level of restriction in research flexibility, time cost, and poor or non-existent incentive structure (Allen and Mehler, 2019). Also, the language barrier plagues OS in Africa as most of the available OS platforms are English, which poses a challenge to science communication (Mwelwa et al., 2020). Some of the solutions to the challenges of OS in Africa have been discussed at different levels by experts and stakeholders, which may include proper funding, stable internet, science infrastructure, leadership, policy development, proper monitoring, and evaluation. In this exploratory review, we analyse the benefits, challenges, and opportunities of OS in Africa. Furthermore, we conduct chronological profiling of the OS platforms and initiatives in Africa. This study serves as an evidence-based informative guide to facilitate advocacy strategies, policymaking, and institutionalization of OS in Africa.

## Benefits of Open Access to Science

The aim of OS is to let anyone access the results of a scientific research or publicly funded research data for knowledge, reuse or innovation purposes. Looking at the various benefits of open data and OS, in January 2021, the Organization for Economic Co-operation and Development (OECD) council, while promoting OS, adopted a revised “Recommendation on Access to Research Data from Public Funding.” The revised recommendation aimed to enhance access to scientific data in order to address global challenges, and at the same time, to advocate for protection of specific data. This is clearly outlined in the European Commission Recommendation 2018/790 of April 25 2018 (European Commission, Directorate-General for Communications Networks, Content and Technology, 2018):

*research data that results from publicly funded research becomes and stays findable, accessible, interoperable and re-usable ('FAIR principles') within a secure and trusted environment, through digital infrastructures (including those federated within the European Open Science Cloud (EOSC), where relevant), unless this is not possible or is incompatible with the further exploitation of the research results ('as open as possible, as closed as necessary'). This could be for reasons, in particular, of privacy, trade secrets, national security, legitimate commercial interests and to intellectual property rights of third parties. Any data, know-how and/or information whatever its form or nature which is held by private parties in a joint public/private partnership prior to the research action should not be affected by these policies or national action plans. (L134/15)*.

**Figure 2:**
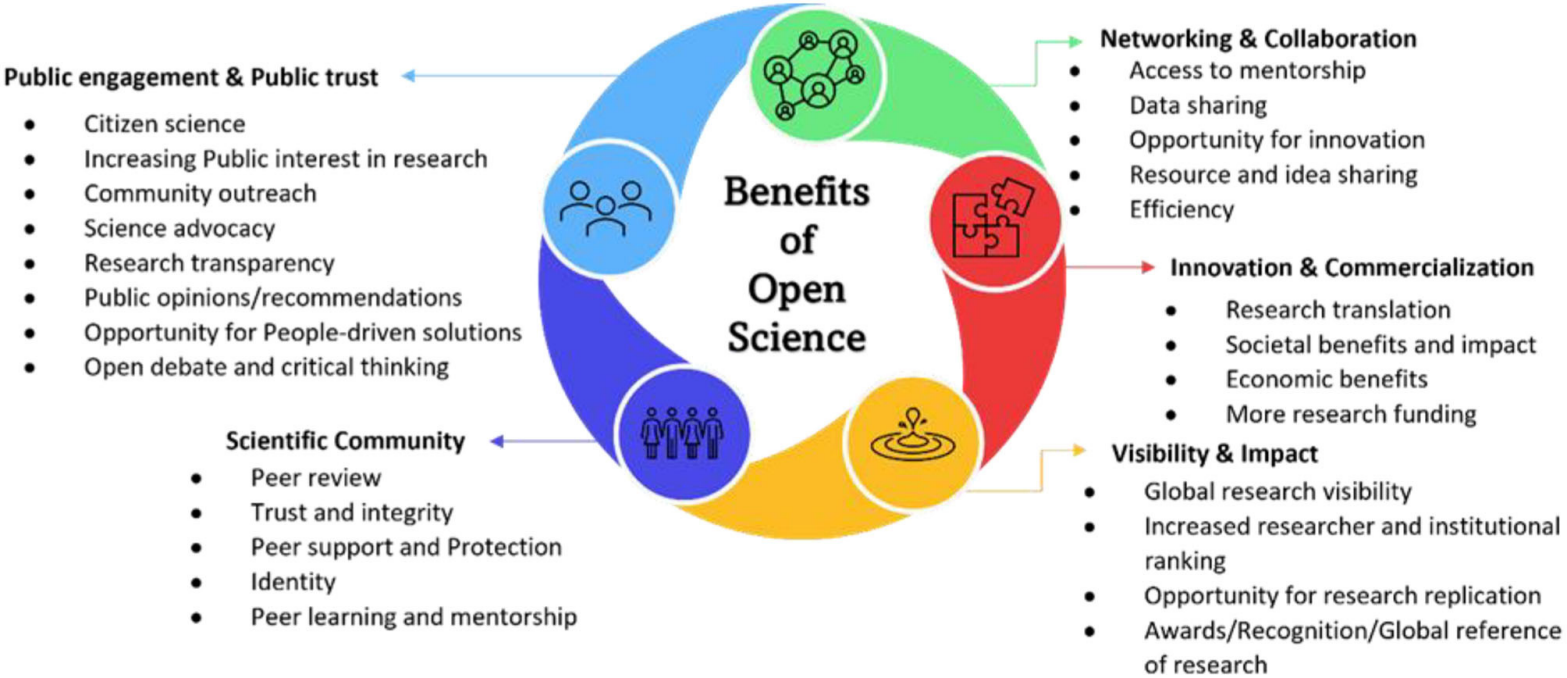
Benefits of open science.

A recent study highlighted the need for shared information to all, citing an example of what the world is currently facing with the COVID-19 pandemic (Paic, 2021). The authors hinted that scientists and researchers around the globe came together and shared their knowledge on the full genome of the coronavirus that could provide a basis for understanding the symptoms, finding ways of treatment, and producing vaccines that may protect people from the virus. The establishment of OA journals to share studies related to the virus was indeed constructive and a way forward in getting all researchers to solve the global issue. Paic emphasized that “the sharing of research data can help accelerate the fight against pandemics and other global emergencies” (Paic, 2021). Evidences have emerged on how data sharing and management both locally and globally has helped to fight the spread of COVID-19 (Budd et al., 2020; Gao et al., 2020). In agreement with this fact, we opine that the relative success being recorded against the COVID-19 pandemic is due to the complete adoption of the different aspects of OS (Figure 1) and the global synergy in doing this. Indeed, there are various benefits to OA or OS. These benefits have been summarized into themes (Figure 2) and further elaborated in the sections below.

### Networking and Collaboration

The first benefit of OS is its ability to promote networking and collaboration among researchers or between researchers and research funders or with other stakeholders. Bezuidenhout et al. (2020) surveyed data sharing by the low/middle-income country scientists belonging to the New Partnership for Africa's Development (NEPAD)-Southern African Network for the Biosciences (NEPAD-SANBio). These scientists believed that data sharing (known as open data) could give them opportunities to build networks and collaboration. For example, in open data, researchers could access and share questionnaires, data, and metadata that could be re-used. Harding (2016), through his work on global health innovation technology models, identified the critical role of an open-source platform that is to enable “re-usable clinical intelligence that can be shared and redistributed in the context of clinical innovation before, during, and after care is delivered” (p. 4). The form of collaboration among the global health community is evident, through the development of mHealth (involving healthcare data exchange *via* mobile phone technology), for effective patient engagement. For this purpose, collaboration occurs in the form of peer-to-peer clinical support. Also, with the mHealth platform, the OS provides a virtual collaboration setting that engages research scientists in quickly sharing knowledge for clinical innovation with their global network. In OA, Besancon and coworkers (Besançon et al., 2021) assert that it allows for peer-reviewing through the open-review principle, where peer-review reports are made open to the public. This is seen as a form of collaboration to help in improving and maintaining the quality of research reports. Another OS element that promotes collaboration and networking is the “open innovation” that promotes interdisciplinary research. Researchers from various fields and even the stakeholders could come together in providing input, designing, producing, and delivering the expected outcomes from the research objectives.

### Public Engagement and Public Trust

OS permits the sharing and transferring of knowledge and scientific data into meaningful information to the general public by giving access to the software and the datasets through OA and open-source platforms. Hudson et al. (2020) opine that “public funders anticipate that research will lead to public benefit” (p. 377). Hence, it is appropriate to engage the public by giving access to the research data and research findings for the purpose of knowledge sharing and dissemination. OS promotes better science-society relationship where engagement is not just referring to the general public but to the various interested parties. This is part of the citizen science initiative where public participation in scientific research is encouraged. Also, the return of meaningful results, from the research carried out, to the public or the funders is considered as part of the social benefits of OS. The transparency in data sharing will also increase public trust in the research conducted. This will be achieved through open evaluation where external involvement for research assessment is made possible. Data and research information that are shared through open system have the chance to be peer-reviewed, annotated, recommended, refuted, discussed, read and taught (Priem et al., 2012). Consequently, this will expand the value of research (Fleming et al., 2021). It will also serve as a means of enhancing understanding, data checking and data confirmation for accuracy (Exley et al., 2015; Shea, 2015) which subsequently establishes the reliability and credibility of the research results that could best be achieved through direct information disclosure in research publications that are accessible to the public, the stakeholders, and the funders (Lakomý et al., 2019). Science communication and public engagement through open-science initiative is important for it allows open debate, initiates critical thinking and allows correcting misinformation from the media (Eagleman, 2013). The public can only become aware of the processes and the complexities of conducting research through the open sharing of knowledge (Lakomý et al., 2019). Science engagements and advocacies must be community-driven and possibly incentivized to increase public trust and participation in science-based decision making processes. In valuing scientific pursuits, public engagements in a form of crowd funding (i.e., contributions from the public) for scientific activities are sought after. Notably, OS is a way of communicating science to the public aiming at developing understanding, changing attitudes, and developing interest toward science, at the same time promoting literacy in science.

### Visibility and Impact

OS in the forms of OA and open data repositories helps in contributing to the visibility of research and in turn leads to greater impact of the research to the scientific community or the society at large. The visibility of research works does not only benefit individual or group of researchers but also their affiliations. The visibility of research works *via* OA helps to showcase and promote the scientists, their affiliated institutions as well as the fund providers. Furthermore, it serves for promotional purposes and also helps institutions to fulfill the requirement of global ranking assessment by publication citations (Momeni et al., 2021). There is an increased probability for others to read and cite a scientific publication with OA, thus enhancing knowledge sharing. One of the impacts from research visibility is that the research could be replicated in other or related contexts. According to Adeyemo and Jamogha (2021), institutional repositories have the role toward enhancing institutional visibility and supporting “scholarly communication among the academic community” (p. 3) especially where the shift from physical print to digital sources has made their work globally accessible. Visibility of research also impacts on the quality of the research being published as scholars are more likely to ensure their work meets certain ethical standards and is worthy of publication and global reference. Advocacy for OA must not jeopardize rigor and quality of publications. Hence, stakeholders must ensure to maintain and keep improving the on current policies that keep the quality of OA publications in check. Institutional repository can be an indicator of institutional quality by displaying works that are of public value (Crow, 2002). Momeni et al. (2021) in their study on the impact of changing publication model from closed to OA, found that the impact factor increases after flipping the journal publications from closed-access to open-access. Their findings reiterated the results of earlier studies (Busch, 2014; Bautista-Puig et al., 2020; Adeyemo and Jamogha, 2021). However, the increase on the impact factor varies across scientific fields (Momeni et al., 2021). In addition, the visibility of research also allows for OERs that could be used for teaching or training purposes which will definitely give a significant impact on teaching, learning, and research development through shared knowledge and practices (Das, 2011; Stagg, 2014; Guo et al., 2015; Bliss and Blessinger, 2016).

### Scientific Community

OS is instrumental in the development of the scientific community. As part of the OS principles, research should be accessible, transparent, re-usable and reproducible. Under this philosophy, a growing scientific community from various disciplines is expected. Data and research information that are available *via* open-source platforms could facilitate scientific collaboration and promote discussions among experts in their respective fields. For example, Taylor and coworkers (Taylor et al., 2017) looked into how OS could benefit Modeling and Simulation (MS) researchers. They asserted that there are various forms of OS that serve as artifacts for MS research (i.e., published research articles, model or simulation program and its execution environment, software, experimentation schema, data, etc.). These artifacts “would be available openly and in a discoverable form” (p. 544), allowing for reproducibility by adopting good open data. To add, OS gives room for verification of research data by other scientists. Beck et al. (2020) who reviewed the benefits of OS on bioassessment, mentioned that “open data products can increase efficiency of the individual researcher and a collective research team by encouraging collaborators to adopt an OS workflow” (p. 6). Additionally, OS and OA initiatives will enhance crowdsourcing for reusability of research data, techniques or methods, by relevant or interested research community of intra- or inter-disciplines. Hetu et al. (2019) based on their study on the impact of open genomic projects, indicated that large-scale databases should be widely accessible to allow advancement of genomic medicine and capacity building in research and development particularly for developing countries where genomic research skills is still growing. They believed that through this accessible databases and OS, researchers or scientists, across the globe could together orient selected projects. They found that international OS project (genomics research, in their case) can make impact on capacity building (of scientific community) through training of researchers, development of research infrastructures, and building of expertise. By having this scientific community through the OS initiative, it helps not only in the capacity building but also in accelerating research (Besançon et al., 2021; Ewers et al., 2021; Kadakia et al., 2021). Kadakia et al. (2021) mentioned that OS “promotes standard processes for sharing protocols and registering studies, reporting and disseminating results, sharing data, biospecimens and code” (p. 1) that allows such data “to be findable, accessible, interoperable, and re-usable to permit independent scrutiny, replication, and follow-on investigations” (p. 1). All these will help the scientific community to accelerate their research. For instance, the COVID-19 pandemic has created urgency for National Institutes of Health (NIH) to set up a research platform that is intended for researchers to share research tools, metadata, and their reports. Similarly, some academic publishers have come together to support preprints to expedite knowledge transfer (Puebla, 2020; Fraser et al., 2021; Hayashi, 2021), and open-access policies to encourage the sharing of information that enable researchers to learn and synthesize from the emerging evidence (Kadakia et al., 2021).

### Innovation and Commercialization

OS initiatives assist in innovating and commercializing research protocols and outputs. Valuable data gathered in the accessible pools of open system, particularly for the scientists, investigators, consultants and researchers, are useful for reproducibility and reusability of research (McKiernan et al., 2016). The reproducibility and reusability are not limited to the data or metadata shared but also to the information related to the research process and procedures, methods and approaches, protocols, models, policies, systems and technologies, cases, etc. These qualities of reusability and reproducibility often times lead to innovation (Kedron et al., 2021). Although Capps (2021) raised a concern over OS becoming a contingency for irresponsible innovation and research misconduct, which necessitates the need for appropriate policies and sanctions to safeguard the future of OS in Africa. Howbeit, we still could not deny the benefits it has to offer with regards to innovation and commercialisation. Again, the recent example from the COVID-19 pandemic that has accelerated the OS practice saw to a cooperative and collaborative work that led to vaccine development. These vaccines have been commercialized with different brand names from Pfizer, Astra Zeneca, Sinovac, Moderna, and Sinopharm. However, “the open commons demand stronger normative principles to support innovative use of new scientific knowledge, but also requires obligations to use it ethically” (Capps, 2021) through open innovation (Wendzel et al., 2017), following this, Granados Moreno et al. (2019) added that OS will expedite innovation by means of partnership and collaborative process that could lead to optimal innovation with minimal economic burden through partnership agreements.

It is evident that all aspects of OS (Figure 1) provide several benefits not only to the researchers but also the public. Among the benefits discussed in this section are networking and collaboration opportunities, building public engagement and public trust, promoting visibility and impact through OA platforms, establishing scientific community, as well as enhancing innovation and commercialization. With these benefits, OS is worth being supported through relevant policies especially in growing economies like Africa.

## Challenges of Open Science in Africa

The primary objective of OS is to increase the value and reliability of scientific output, increasing efficacy and spurring discovery and innovation (Nosek et al., 2015; Heuritsch, 2020). Multiple mechanisms are employed to achieve this objective including deliberate institutional policies, infrastructure and relationships that promote OA publications, open data and scientific resources as well as removal of restrictive intellectual and other proprietary rights (Ali-Khan et al., 2018). The requirements to drive these mechanisms are enormous and laden with several challenges. The challenges hampering the adoption and development of OS are even more pronounced in resource-limited environments like Africa (Mwelwa et al., 2020; Mwangi et al., 2021). Directly or indirectly, many of these barriers may be associated with lack of adequate funding for education/research which is reported to fall below expectations in many African countries (Teferra and Altbachl, 2004).

Due to insufficient funding, several African researchers lack state-of-the-art facilities which are available to their counterparts elsewhere (Kokwaro and Kariuki, 2001; Yusuf et al., 2014). This directly impacts OS which relies heavily on technology and skills. African researchers are unable to undertake OS projects, having to work with limited resources, thereby reducing the propensity for quality and credibility which are the hallmarks of OS. The cost of disseminating research is another challenge as in most cases such cost have to be incurred personally by the researchers due to lack of grants which could have covered research publications (Ahinon and Havemann, 2018). This has informed the decision by several publishers to give varying amounts of discounts on article processing charges or outright waivers to researchers from low and middle income countries. However, these seems not to be enough as the prices after discount are still unaffordable since these charges are in foreign currencies which have higher values than most African currencies (Ezema, 2011). Paucity of research sponsorship sets the stage for several other impediments to OS in Africa such that if addressed could revolutionize OS on the continent.

Relatedly, lack of infrastructure, availability of tools, and processes that aid OS infer low skills to champion OS among African researchers. Authors have noted the low internet penetration in Africa compared to the developed countries, and even when available, the speed is typically slower (Steiner et al., 2005; Les Cottrell, 2013). Cyber infrastructure for instance enables investigators to cope with the data volume, provide effective data interfaces and visualization and utilizes more powerful algorithms to extract more information from these data sets (Ramachandran et al., 2021). Consequently, availability of infrastructure and capacity building on research design, data entry and the use of cyber platforms is indispensable to the advancement of OS in Africa.

Lack of deliberate policies and legal frameworks promoting OS from governments, institutions and funders prevents the advancement of OS in Africa (Onie, 2020). In the European Union for example, there is a well-coordinated policy and programmes on OS (European Union, 2017). Policies must be balanced and focused on how to navigate some of the potential barriers of OS policies such as privacy, trade secrets, national security, legitimate commercial interests and intellectual property rights of third parties. The availability of policies together with the provision of funds has improved OS in Europe and has placed it as one of the leading regions for OS (Leonelli et al., 2018). Relevant policies with strong legal frameworks can be institutionalized in Africa and tailored to meet the localized realities of the challenges OS face in Africa.

The lack of OS awareness is an additional hindrance to OS among African researchers (Teferra and Altbachl, 2004; McKiernan et al., 2016; Mwelwa et al., 2020). Many are unfamiliar with OS and/or its ramifications; as such they have not bothered to develop the requisite skills that enable the practice of OS. Some African researchers who are well informed about OS are reluctant to practice it due to the lack of incentives for OS practices (Allen and Mehler, 2019). Typically, researchers employing traditional methods get results quicker and publish faster unlike OS methods which take longer due to the complexities of transparency. With the pressure for academics to “publish or perish”, traditional researchers have greater possibilities for faster career progression because of the time demand of OS practices (Allen and Mehler, 2019). With low motivation and less recognition given to OS practitioners in Africa, researchers are further discouraged to fully practice OS. These researchers are usually keen to access quicker routes to publish their research to further their careers and in many cases encouraging unethical and illegal practices to get desired results. Such practices embolden corruption in the academia in a direct antagonism to the integrity and quality which OS seeks to entrench (Nosek et al., 2015; Heuritsch, 2020).

OS requires partnerships and collaborations between stakeholders including government, academic and research institutions, research funders, researchers, libraries, publishers, information and communication technology experts and end users of the research outputs (Kennedy and Ruttenberg, 2019). In the absence of these alliances, advancing OS in Africa remains challenging because these interdependencies are complementary in achieving OS (Krishna, 2020).

## Opportunities and Future Considerations

### The Strides Toward OS in Africa

The OA movement in Africa is gradually growing. As at 2015, over 500 OA journals published in Africa are captured in the African Journals Online (AJOL) and Directory of Open Access Journals (DOAJ). Meanwhile, 125 OA digital repositories in Africa are registered in the Directory of Open Access Repositories (OpenDOAR) while 18 OA policies from the region are listed in the Registry of Open Access Repository Policies and Mandates (ROARMAP). Ever since, a lot more African researchers also publish in international OA journals. Some African institutions are on the lead and have taken initiatives to boost OA movement in Africa. For instance, Stellenbosch University offers on-site trainings and shares valuable materials online to new OA repositories while the Academy of Science of South Africa (ASSAf) in partnership with UNESCO Cluster Office in Southern Africa offers training to OA journal publishers in the region (Academy of Science of South Africa, 2022).

International organizations like Electronic Information for Libraries (EIFL) and International Network for Advancing Science and Policy (INASP) support libraries in the region while the Irish African Partnership for Research Capacity Building (IAPRCB), joins several universities in Ireland, Malawi, Mozambique, Tanzania, and Uganda to develop a harmonized approach to research capacity building through its OA repository. The International OA Week has been made annual events in some African research institutions and helps in raising consciousness among academic communities of the region about the benefits of OA.

In addition, the Southern African Regional Universities Association (SARUA), representing 66 public universities in Southern Africa, published a research report on Opening Access to Knowledge in Southern Africa, recommending OA as a potential strategy for Africa. To some extent, all major stakeholders—researchers, research administrators, policy makers, journal editors, publishers, librarians, OA experts, students and general public—have started to realize the benefits of OA and seem to be making efforts, albeit little, to implement OA projects in the region.

UNESCO had shown their readiness to work with African countries willing to work toward national OA policy and also called for training centers to build capacity and expertise on OA ideas and structures. In 2015, UNESCO, Network of African Science Academies (NASAC), Royal Netherlands Academy of Arts and Sciences, African Academy of Sciences (AAS), Kenya National Academy of Sciences, and Kenyan Ministry of Education, Sciences and Technology jointly hosted a consultative meeting on OA for Africa in Kenya bringing together about 45 top policy makers and expert representatives of 20 countries of Africa providing intervention for research and development in Africa.

The recent landmarks on OSs created the opportunity for OS discussions in Africa in recent times. The Council for the Development of Social Science Research in Africa (CODESRIA) hosted her conference in 2016 titled “Electronic Publishing: OA Movement and the Future of Africa's Knowledge” in Dakar, Senegal where opportunities and challenges to the OS movement in Africa were discussed. This led to a Dakar Declaration on OS in Africa where all the signatories agreed to promote and support OS across Africa by organizing events on OS which will target both students and researchers. To foster scientific innovation and capacity to contribute to global scientific research output, African countries should be provided with virtual high-tech laboratories under an open license in addition to the standard OA materials such as course materials, textbooks, multimedia applications etc. This trend is beginning to emerge as there are online scientific laboratory initiatives in Africa that will boost the commitment to OS as an academic practice. Eighteen African governments have established the African Virtual University Project (AVU), a leading eLearning Network in Africa with the vision to meaningfully increase access to quality higher education and training through the innovative use of information communication technologies (ICTs) covering several scientific disciplines. Similarly, Africanfossils.org has also been established as a free online virtual lab for scholars to explore and interact with fossil collections under the Creative Commons Attribution-Non Commercial-Share Alike License (Canton, 2021).

The LIBSENSE initiative launched in 2017 has been building a community of OS practitioners and making headway for the adoption of OS services and structures in Africa. The initiative was led by West and Central African Research and Education Network (WACREN) in partnership with a number of other organizations with the aim to advance OS in Africa through strengthening and expanding services at the institutional, national and regional level (Abbott et al., 2020; WACREN, 2021). In 2021, a virtual workshop was co-organized by LIBSENSE partners, EIFL, WACREN, UbuntuNet Alliance, the Arab States Regional Education Network (ASREN), Confederation of Open Access Repositories (COAR), AfricaConnect 3, OpenAIRE, and GÉANT, which showcased the current national level activities on OS policies, repositories, community building and coordination in 15 African countries. As a follow up, three working groups were set up, on (1) OS policies, governance and leadership; (2) OS infrastructure—OA journals, repositories for publications and data and open discovery services; and (3) capacity building—communities of practice and training. In addition, this workshop set up region-specific and language-specific discussions in Arabic (North Africa) and French (West and Central Africa) working groups who were charged with co-developing guidelines, support and training materials (Abbott et al., 2020; WACREN, 2021).

LIBSENSE also started a series of open community calls, titled “Co-designing collaborative free and open source-OA publishing infrastructures”, organized by WACREN, EIFL, and the Coko Foundation, with African journals and books editors and publishers, researchers, librarians and tool builders. In these calls, they discussed needs and tools for OA scholarly publishing in Africa; what open source tools and services for publishing books, journals and textbooks are currently in use, and the training and support needs (Abbott et al., 2020; WACREN, 2021; Kuchma, 2022). LIBSENSE recognizes that OS in Africa, with respect to diversity and sustainable development, can be best realized through localized, yet interoperable, infrastructures—rather than being subcontracted to private industry or external organizations. These services will be able to more directly answer to the necessities of African research communities, and also contribute to building local capacity and knowledge around OS (Abbott et al., 2020). It is important to note that the UNESCO OS Partnership has put OS on the national agenda of several African governments. Taking advantage of this strategic opportunity, LIBSENSE has begun to work with several African countries that are committed to advance OS policies, infrastructures and services to develop African National OS Roadmaps that can then be piloted in other African countries (Abbott et al., 2020).

The above discussions illustrate a trend in OS efforts in recent times. The high tendency to work in isolation by African scientists and scientific organizations has implications in the effectiveness and efficiency of science systems, thus limiting the needed collaboration to address the complex problems of OS in Africa (Bezuidenhout et al., 2017). The recent endorsement of the Africa Continental Free Trade zone Agreement among member states of the African Union (AU) and the AU's efforts toward actualizing United Nations Sustainable Development Goals, could afford the stimulus needed for development of an inclusive and strong OS initiative in Africa (UNSDGS, 2015). We hereby identify and discuss the opportunities and future considerations for OS in Africa below.

Globally, OS movement has been witnessing an unprecedented increase in its embrace. Although weakly implemented in Africa according to the UNESCO report in 2015 (Mwelwa et al., 2020), there is a gradual and steady adoption of this movement currently with variation between the English and French-speaking African countries (Ahinon and Havemann, 2018). Several OS initiatives have been established in Africa or for African researchers under the various elements that constitute the concept of OS. For example Open Science in Haiti and Francophone Africa (SOHA) project and African Open Science Platform (AOSP) are under OA (advocacy and publishing) initiatives while Africa Open Science and Hardware (AfricaOSH) is an example of an initiative or platform for OS hardware. We highlighted in this review some major OS platforms or initiatives in Africa (Table 1).

### Open Science Policy and Policy Makers

Government institutions in Africa have adopted an open government charter that requires them to open some of their data assets, in a way that many national statistical offices now collaborate internationally in developing open data practices (Nordling, 2015). Despite the 18 OA policies from Africa registered in the Registry of OA Repository Policies and Mandates, Africa is still in shortage of functional policies and policy making bodies on OS. The areas of OS where policies are required include funding, research data management, IP, and copyright (Mwelwa et al., 2020), It is important that IP protection is well-balanced to protect the rights of originators without reducing the chances of innovation (Mwelwa et al., 2020). Again, Africa has been criticized for poor performances with implementation of some global policies. African states accept policies as a working document but usually do not drive the implementation due to the lack of strong systems, stable leadership and integrity. Irrespective of reports of suboptimal success with several other African policies, it is necessary that Africa makes an evidence-based attempt to respond to OS campaigns with a dependable and unifying policy. There is lack of key policies that are necessary for the advocacy and industrialization of OS in Africa both at the national and institutional levels. The enactment of regulatory policies or regional framework for OS is paramount, from the acquisition of data, data usage, management, publication, translation, and re-use.

There are key organizations in Africa (both private and government-owned) that work in the field of OS and have some form of internal frameworks to fulfill their set objectives (Table 1). However, some of these organizations may not be able to single-handedly unite Africa on an operational model for OS in Africa. The question to ask is, “do these existing organizations in Africa have such potential and capacity to drive the conversations around a holistic OS policy for Africa?” The African Union Commission (AUC) through her department of Science, Technology and Innovation had made a recent attempt to bring experts and stakeholders together through the African Regional Multi-stakeholder Meeting on OS in 2020 (UNESCO, 2020). While this is a good first step, it is imperative that this be sustained. It is not clear if there is a roadmap, timeline, and strategy for achieving the set goals declared in the regional meeting. More so, there may be a need to involve or get technical support from global OS platforms like International Science Council (ISC), UNESCO and other collaborators in future conversations to share their experiences for early identification of potential pitfalls. There is a need to set up a special caretaker committee, for example within the AUC to organize, supervise, implement, monitor and lead the development of OS policies for Africa. However, a strong leadership system within the AUC and African countries is needed to drive the sustainability of the above-mentioned initiatives.

The capacity for Africans to fully exploit the opportunities presented by digital revolution that could drive innovation and development on the continent would be greatly enhanced by a strong, multi-state OS system (Boulton et al., 2020). In this digital age, Africa needs to explore digital technology for the continent to economically benefit from the fourth industrial revolution (Ndung'u and Signé, 2020). OS stakeholders in Africa need to take advantage of Africa's increasing interest in Internet and other digital technologies (Kende, 2021). Hence, technology must be at the forefront of any policy being developed by Africa on OS. A good place to start in the consideration of OERs is to have clear and effective policies on IP right and copyright. For example, concerning intellectual capital, a clear policy would plainly lay out the corresponding rights of the institution, its employees, students, or contractors, who are involved either directly or indirectly with sharing materials. In policy negotiations, it is important to consider the relative benefits of making flexible copyright policies that spontaneously apply open licenses to contents except for serious reasons to retain all-rights reserved copyright over such contents. Concurrently, any of such policies should still make it easy for anyone to invoke all-rights reserved copyright whenever necessary. Human resource policies should be developed where non-existent or reformed by updating of costing/resourcing and performance management systems so that African scientists can be rewarded for: (1) time spent in educational resources development, (2) using resource-based learning (when it is more effective), (3) using available materials with similar content (when it is more cost-effective) than producing a new one, and (4) sharing intellectual capital through global networks to improve resources, personal and institution's profile.

The AU's Agenda 2063 (AU, 2015) and the United Nations Sustainable Development Goals (UNSDGs, 2015) will be far from being met in Africa if OS is not prioritized through a working policy for Africa. Again, some pre-existing policies, habits and processes that had earlier been helpful are now obvious inhibitors of innovation, and need to change (Mwelwa et al., 2020). Africa needs to be involved critically in the discussions of private sector monopolization of scientific data by the ISC (International Science Council, 2019) as they are major preys to this. African policy makers must bear in mind the critical areas to focus in their policy review or engagements. These have been well-outlined by Mwelwa et al. (2020):

“*managing, curating and using large and diverse data volumes, developing the incentives, methods and standards for data sharing, maintaining security against malign interventions, ensuring the preservation of ethical standards, developing the systems and software to undertake all these tasks and keeping abreast of the rapidly evolving state of the art in data science”*.

Scientists need customized research systems to do robust and transparent science especially in Africa where young researchers are working to build OS practices from the scratch. The needs of African science communities are different from those that are part of more developed research systems. Hence, there is need to drive sustainable policies and their implementation that will reflect each country's needs and ensure consistent growth of OS in Africa (Onie, 2020). An important question to ask regarding OS policy development is whether OS policy should be institutional specific or whether this could be standardized across African institutions.

### Funding for Open Science

Funding for OS in Africa remains untraceable, unstable or unsustainable. There is neither funding documented for the African region nor African organizations documented as funders in the UNESCO's global OA portal funding mandates for OA for world regions. There is a need to apply funding toward retooling universities for research. For example, Nigerian librarians struggle to acquire current scholarly literature and modern technology for their libraries because of constant budget cuts (Okere, 2020). Dedicated and reliable funds are necessary to sustain scientific research in higher institutions. Funding is necessary for the provision of support staff, investment in research travels and establishment of procedures for data collection, grants management, and ethics. These provisions would enable effective teaching and research in science.

It is unclear how many African institutions have self-owned OERs and repositories (Bezuidenhout et al., 2020). These resources are either occasionally accessible or non-existent in most African institutions perhaps because of the low funding for African universities and research institutions (Bezuidenhout et al., 2020). It should become compulsory to establish universities with these resources being considered as part of its accreditation criteria to create a systematic funding for OS in African institutions.

Openness in research demands an e-infrastructure that will expedite information sharing. Therefore, there is a need for accessibility of reliable internet connection that can enable data sharing, especially for large datasets usually seen in bioinformatics and imaging research. In addition to a reliable internet, storage and high-performance computing capabilities are also essential (Maphosa, 2019). In a poor-resource setting like Africa, e-infrastructure to support OS is inadequate (Maphosa, 2019). However, the development of National Research and Education Networks (NRENs) in Africa is advancing speedily (Foley, 2016). NRENs provide internet infrastructure and services to support research and educational activities within a country (Dyer, 2009). The ICT infrastructure provided by NRENs is key in facilitating OS practices, starting with the open scholarly literature through institutional repositories in African universities and research institutions. Again, NRENs offer OERs and facilitate research collaborations between local and international scientists (Foley, 2016).

The major funders for OS in Africa include African Development Bank, African Union, research and health ministries, heads of state, Gates Foundation, Chan Zuckerberg Initiative, World Bank, and others who have given considerable support to African-led projects and networks that reinforce research. Examples include the AOSP (funded by the National Research Council of South Africa) and the Alliance for Accelerating Excellence in Science in Africa (AESA)—partnership of AAS, NEPAD Agency with US$5.5 million initial seed funding from the Bill and Melinda Gates Foundation, the Wellcome Trust and the UK Department for International Development (UkDFID). These funders provide not only funds for future infrastructural development, but also expertise, and contacts to international expertise and national/regional governance stakeholders (Havemann et al., 2020).

In the face of inadequate funding for science in most African countries, it is important that countries utilize free open source hardware (FOSH) to be able to avert the cost implications of scientific equipment or to reduce them drastically (Maia Chagas, 2018). Maia Chagas (2018) has documented all the notable FOSHs for scientific research and education with the associated links. Also, it is important that OS actors in Africa: (1) identify ongoing projects and funders addressing infrastructural reform on OS, (2) approach funders and governments directly, and (3) create a record of grants on OS, to co-apply and consolidate for overlapping activities to maximize funding opportunities. Governments and international bodies can adopt the performance based financing for OS initiatives at any level to be able to attract and sustain effective and efficient initiatives for African countries (Sieleunou et al., 2017). It will be waste of scarce resources for lower-income African countries to fund research without expected scientific rigor and integrity. Thus, funding policies should not just be targeted at increasing output but also intended to improve relevance, transparency, and scientific rigor, especially if research outputs are geared toward being useful for decision-making in Africa. Governments should provide the motivation and training resources needed for people to imbibe the policy changes. It is believed that when there is an increased impact of OS funding through increased innovation or productivity, countries will be more willing to commit more to financing OS initiatives. Overall, “investments should generate a virtuous cycle in which long-term changes in research output yield more government and international funding” (Onie, 2020).

### Advocacy and Incentives

The sustainable culture of OS can thrive in Africa if there is adequate institutional or individual advocacy plans toward OS. First, we need to start a global advocacy for the relevance of the scientific content emanating from Africa (Pennisi, 2021). The exclusion and neglect of science done in Africa and by Africans in the global scientific decisions and policies contributes to the disinterest of African researchers from OS practices (Mwelwa et al., 2020). Such unconscious bias affects the understanding of the natural world, and makes it more difficult for researchers from Africa to operate effectively (Harris et al., 2017). Getting included in systems where OS is already established will boost the uptake level of OS by African researchers. Following the authors' experience and peer reports, there are none or few Africans in the editorial boards of notable open access science journals. This inequality may contribute to the understanding of science-based issues in Africa, and their acceptance for publications (Onie, 2020). Again, it is a common experience with the authors and their peers on increased non-acceptance of scientific articles from African researchers by top scientific journals on the bases of being insignificant to the wider readership or simply because of using a traditional method, usually still in use in Africa (Onie, 2020). A study presented US researchers with identical abstracts and observed that the researchers were more likely to recommend the article to a peer if its authors were listed as being from the United Kingdom than if they were from Malawi (Harris et al., 2017). Advocacies against these biases will spike the confidence of African researchers toward OS practices. Journals should take the lead in reducing under-representation, while maintaining scientific rigor, and authors should explicitly describe their study populations ahead of time, and not generalize their findings beyond the study population without any good justification. Double-blind reviews have been used to tackle the positive bias experienced by prestigious institutions or authors (2015) while the use of Open reviews could reduce potential bias against studies from African researchers (Carroll et al., 2017; Onie, 2020). Open publication systems will give the reputable non-African journal publishers the opportunity to share ideas on publication standards especially relating to OS policy which African researchers and publishers could adopt. More recently, we have also experienced special issues focused on the under-represented populations. The sustenance of such cultures will not only encourage equity and remove bias from global interpretation of scientific data, but also give more opportunity for OA publications amongst African scientists.

Strong advocacies against some academic practices which discourage open and rigorous science in Africa is necessary to achieve a quickened OS culture in African institutions. Many African institutions judge their faculty members according to Western standards, including publishing in “prestigious” journals. The pressure to publish at all costs to meet certain promotional criteria is one of the biggest challenges to creating credible scientific output from Africa (Rawat and Meena, 2014).

Again, the use of metrics for faculty appraisals should only be applied if they seem useful to the overall goal for science—knowledge accumulation for the greater societal good. For example, AAS open research platform does not utilize the impact factor metric system characteristic of many journals as they describe it as flawed. The AAS Open Research model is part of advancement in scientific publishing that berates the use of such measures. Individual articles published in the AAS platform displays article-level metrics as and when applicable, such as Altmetrics; PubMed citations for articles that have passed peer review; and the number of views and PDF downloads on AAS Open Research and in PubMed Central.

It is proper to introduce an incentive system to encourage more African researchers to easily adopt OS. Some incentives that should be supported at all levels includes provision of research grants, publication grants, travel grant, training grants and also rewarding OS practice during promotional assessments. However, it is essential to know that the best ideas to improve science today may become less useful in the future. For example, China recently stopped its cash-for-publication system after realizing its impact on the quality of publications. This is a proof that sustainable change to good behavior cannot be achieved when people are indirectly incentivized to do the opposite (Mallapaty, 2020). The reward system should encourage research cultures that can guard against harmful practices and lay down a good strategy for OS advancement in Africa (Mallapaty, 2020).

### Collaboration and Networking

Collaborative research in sciences is relatively low in Africa and needs to improve (Onyancha and Maluleka, 2011; Pouris and Ho, 2014). Scientists from growing research cultures like Africa should be encouraged to join societies and conferences hosted in countries with more openness in science, to create the mutual scientific exchange necessary for OS behaviors. There is a need to acknowledge and confront the isolation of African scientists from opportunities for international collaboration. Inadequate funding and travel restrictions in many countries in Africa reduce the opportunities for networking, international collaboration and lead to more isolation of African researchers (Ochola and Gitau, 2009; Ranganath, 2017; Kasprowicz et al., 2020; Marincola and Kariuki, 2020). It is necessary that efforts to make space for African researchers be focused on empowerment and based on mutual respect, rather than taking control of their systems (Minasny et al., 2020). Strong partnership and collaboration policies must be in place to ensure collaborations between African researchers and researchers from the developed countries are true partnerships (Minasny et al., 2020). More collaboration amongst African researchers with OS requirements should be initiated and incentivized by OS stakeholders. The multilingual nature of Africa creates opportunity for collaborations that are not limited by language bias. Such initiatives should be sponsored by scientific societies and other stakeholders of OS at all levels to encourage the deepening of OS practices and entrenching the OS culture amongst African scientists.

### Training and Capacity Building

The question to ask as Africa embraces advancements in OS is “who needs to be trained?” This should be informed by research and not by assumption. We posit that every length and breadth of the stakeholder chain in African OS system may require some form of training or the other (Onie, 2020). Having a few persons or organization which shows some degree of expertise may not be enough for the wholesome intervention needed in Africa. Stakeholders must first assess the training needs of open system drivers in Africa and her beneficiaries to ensure its effective implementation, usage and replication. Awareness must be created on OS tools and skills for OS practice to become easily adoptable. The European Commission OS Skills Working Group recommended that researchers should be sensitized and trained on OS practices that ease the practice of OS such as open research, OA, open education, open data, open peer review, and citizen science. Capacity building to enable effective use of OER should involve: (*1) supporting policy-makers and heads of institutions to understand the key elements necessary to create supportive policy environments, develop materials, use technology, and conduct research; (2) identifying best-practice examples of use of OER and facilitating institutional visits, so that participants have an opportunity not only to observe effective use of OER in practice but also to start developing support networks and communities of practice* (Organisation for Economic Co-operation Development, 2007).

The essential skills needed for institutions to effectively utilize OER include:

- OER advocacy and promotion skills.
- Knowledge on content licensing legal framework.
- Skill in business model development, course development and programme design.
- Technical know-how and network management.
- Expertise in monitoring and evaluation.
- Skills in effective curation and sharing OERs.
- Research and communication skills for information sharing (Organisation for Economic Co-operation Development, 2007).

To aid training in OS, free online resources should be readily available. In recent times, there has been a surge in written materials, YouTube videos, and mass open online courses in different languages (Onie, 2020). Beyond training on OER, African scientists need competency training on specific areas of science especially areas where expertise is generally lacking or where scientific infrastructure is limited to improve their global participation in those areas. Training in good scientific practices will position African scientists to be more critical and adopt practices that improve the integrity of their work. It will also allow them to add their diverse voices to the on-going multifaceted debates on OS, paying attention to the benefit of science to the society, locally and globally (Onie, 2020). Training should also boost scientists' career trajectories especially now that institutions are beginning to seek for evidence of OS practice as criterion for recruitment (Onie, 2020). Institutionalizing OS in Africa is critical to capacity building for the next generation scientists in the region. OS is needed not just for OS-related capacity building but also for easy access to technological and scientific skills which are lacking in Africa, that are necessary to drive the scientific innovations and development in the region.

## Study Limitations

This study might have missed out some important articles on OS published in other languages other than English, considering the multilingual nature of the African continent. Again, we could not access a few non OA articles at country or organizational level which may also be useful to our evidence synthesis. The evidences available in literature may not be reflective of the true situation in some countries of Africa or may have since changed especially as there are a few or no information on open science practices in many African countries. However, we carefully executed the literature selection of this exploratory review and ensured contextual interpretation of our findings to enhance the usefulness of this study as an advocacy tool for OS in Africa.

## Conclusion

**Figure 3:**
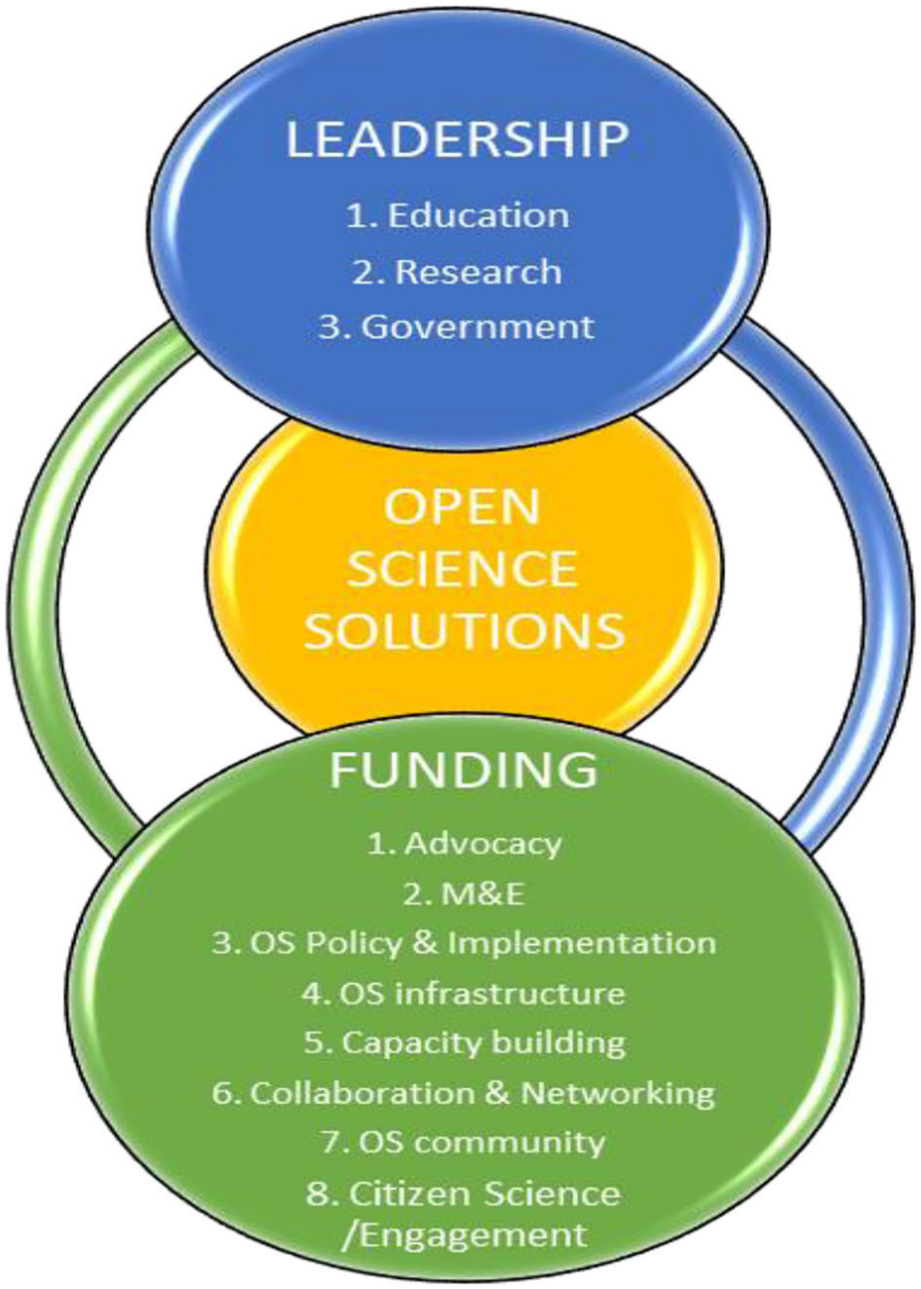
Conceptual framework for creating open science solutions in Africa. M&E, monitoring and evaluation; OS, open science.

In this exploratory review, we critically analyzed a reemerging issue in Africa—OS—occasioned by the COVID-19 pandemic. We highlighted OS benefits, OS platforms, challenges, and opportunities in Africa. OS offers several benefits to the development of science in Africa including but not limited to sharing of scientific data. It provides opportunities for all to work together at the same time building understanding of research procedures, practices, and findings. OS stakeholders need to promote, utilize, and upscale the OS platforms and initiative highlighted in this study and only then can they benefit from OS as themed above: networking and collaboration, public engagement and public trust, visibility and impact, scientific community and, innovation and commercialization. Some of the major challenges that plague OS in Africa were highlighted in this review but the lack of funding for science seems more critical as it directly impact other challenges. We have created a conceptual framework for creating OS solutions in Africa following the evidences generated from the literature on the challenges of OS in Africa (Figure 3). This is a two-prong framework that shows the non-sequential overlap and interdependence of funding and leadership as the pivot for creating thriving OS systems in Africa. As discussed above, funding is key to delivering many OS initiatives and strategies; and these OS solutions are to be steered by dependable leadership system built across education, research and African governments at different levels (Figure 3). It is important to note that there are inequalities in the practice of OS in Africa even amongst African countries, individual researchers or organizations as a result of varying challenges. Hence, there is need to conduct both qualitative and quantitative research to understand organizational and individual perspectives of OS practice especially concerning challenges and choices of OS practice. This will enable the creation of a more sustainable advocacy or implementation that works for each country. Policy making in OS must take into consideration the context of each country's challenges to maximize opportunities in its implementation. OS still remains a significant contributor in solving global problems, and thus a potent channel for Africa's development. Hence, institutionalizing OS in Africa should be on the forefront of science stakeholders in Africa more than ever before, especially due to the current pandemic.

## Author Contributions

IO and SM: conceptualization. IO, SM, TC, ZH, EU, and KC: methodology, data curation, visualization, validation, resources, writing—original draft, and writing—review and editing. IO: project administration. All authors contributed to the article and approved the submitted version.

## Conflict of Interest

The authors declare that the research was conducted in the absence of any commercial or financial relationships that could be construed as a potential conflict of interest.

## Publisher's Note

All claims expressed in this article are solely those of the authors and do not necessarily represent those of their affiliated organizations, or those of the publisher, the editors and the reviewers. Any product that may be evaluated in this article, or claim that may be made by its manufacturer, is not guaranteed or endorsed by the publisher.
