# Supplementary material for: Institutionalizing Open Science in Africa: Limitations and Prospects
Source: Front Res Metr Anal. 2022 Apr 15;7:855198. doi: 10.3389/frma.2022.855198 (PMC9051436; doi:10.3389/frma.2022.855198)
Supplement: Supplementary file 1 [file Data_Sheet_1.docx]

**Literature Search Strategy**

Our data sources included a review of relevant documents and publications with a specific interest in Africa. We searched articles using different academic databases, including Medline, Web of Sciences, Google Scholar, PubMed, and Science Direct. We also searched Google, government, and relevant organizations' websites for communications, reports, policy briefs, and other kinds of publications on OS at the organizational and government level. These databases were purposively chosen as they encompass a wide range of studies on OS. The keywords utilized for the literature search include 'Open Science', 'Open Access', 'advantages', 'benefits', 'Open Science initiatives’, 'Open Science platforms', 'challenges', 'opportunities', 'solutions' and 'Africa' in line with Medical Subject Heading (MeSH) terms. We diversely combined these keywords, considering their synonyms, variant spellings (American and British), generic names, quotation marks, and truncations. Also, Boolean commands of 'OR' & 'AND' were utilized in linking the keywords. We avoided applying date restrictions to the search to get every available evidence on the subject matter.

All the resulting articles from academic and non-academic database searches reflecting the Medical Subject Headings (MeSH) for OS were included for review. Articles were independently screened using their titles and abstracts sequentially. Afterward, each article was reviewed to exclude irrelevant articles. Only articles published in the English language with a focus on OS in Africa were included. Furthermore, a snowball search was utilized to iteratively scan the reference list of included articles for a further selection of published studies on OS in Africa. Several articles were reviewed to critically analyze and generate insight into OS in Africa's context, specifically the benefits, challenges and opportunities of OS. Findings were structured into different themes of focus for the development of the different sections of the study. As posited by Braun and Clarke (2006), thematic analysis was utilized in analyzing Africa's OS benefits, challenges, and opportunities in this exploratory review. Data relating to OS platforms and initiatives in Africa were extracted in a table using Microsoft Excel, while components and benefits of OS were modeled using Figures. This review required no ethical approval.
